# Assessing universal considerations in infant mortality across the globe: A descriptive observational study of sudden infant death syndrome knowledge and reduction coverage on YouTube

**DOI:** 10.34172/hpp.43055

**Published:** 2024-10-31

**Authors:** Aysha Jawed, Amy Hess, Molly Rye, Catherine Ehrhardt

**Affiliations:** ^1^Department of Pediatrics, Johns Hopkins University School of Medicine, Baltimore, MD, USA; ^2^Department of Pediatric Social Work, Johns Hopkins Children’s Center, Baltimore, MD, USA; ^3^Department of Pediatric Nursing, Johns Hopkins Children’s Center, Baltimore, MD, USA

**Keywords:** Cot death, Crib death, Health communications, Health promotion, Infant mortality, Social media, Sudden infant death syndrome

## Abstract

**Background:**

Sudden unexpected infant death (SUID) remains one of the leading causes of infant mortality worldwide and is largely driven by sudden infant death syndrome (SIDS). Although SIDS has received coverage and examination of content spanning Instagram, Facebook, and Twitter across the published academic literature, there is no study to date that has examined SIDS related content on YouTube.

**Methods:**

This descriptive observational study was conducted from December 2023 through January 2024 and sought to describe the sources, formats and content covered across the 100 widely viewed videos pertaining to SIDS on YouTube.

**Results:**

The majority of the videos published were by organizations (N=64) including healthcare systems, the American Academy of Pediatrics (AAP) and police departments. Several of the widely viewed SIDS-related content was disseminated by professionals (N=42). Multiple videos presented content on the symptomology pertaining to SIDS as well as contributing environmental risk factors. A wide range of resources were depicted as SIDS reduction measures. Notably, there was substantial emphasis on SIDS reduction postnatally across the widely viewed videos. There was limited representation of content on SIDS awareness and reduction outside of the United States.

**Conclusion:**

Clinical, public health, and organizational implications and recommendations are presented to inform future targets for intervention that can harness findings from this study on widely covered and uncovered content to address the totality of risk factors for SIDS. Future directions in health promotion across the SIDS reduction landscape are also reviewed to account for digital spaces globally, thereby contributing towards reducing infant mortality worldwide.

## Introduction

 Sudden Unexpected Infant Death (SUID) is one of the leading cause of infant mortality worldwide and is largely driven by sudden infant death syndrome (SIDS).^1^ As defined by Krous et al, SIDS involves the unexplained death of an infant, oftentimes from environmental risk factors postnatally as well as prenatal factors that remain unaddressed during time of pregnancy.^2,3^ Furthermore, SIDS accounts for the majority of SUID cases globally.^1^ Based on the most recent study assessing the global burden of SIDS, there are nearly 28 000 SIDS related deaths estimated worldwide as of 2019.^4^

 There are a host of evidence-based modifiable and nonmodifiable risk factors that can heighten an infant’s risk for SIDS. Some of the most prevalent ones include an unsafe infant sleep environment (e.g. loose bedding, choking hazards, prone sleeping), tobacco, substance and alcohol use and exposure among caregivers of infants, thermal conditions, low birth weight, prematurity and so many more.^3,5^ In addition, the interplay of these risk factors (e.g. co-sleeping with tobacco, alcohol, and substance use) also elevate an infant’s risk for SIDS and SUID.^3^

 The United States has taken a visible presence in addressing a host of these risk factors at community and institutional levels. For example, the American Academy of Pediatrics (AAP) has published national infant safe sleep guidelines that are publicly accessible to the healthcare community worldwide. These guidelines extend to include recommending prenatal care to initiate addressing a range of environmental risk factors on a continuum from time of pregnancy to post-birth.^5^ The AAP has continued to update these guidelines based on evidence-based findings from research on current and additional contributing risk factors for SIDS and SUID. These guidelines include a host of recommendations to reduce environmental exposures for infants and optimize conditions in their sleeping environment as well as promote continued research on addressing systemic disparities and inequities surrounding SIDS and SUID. In addition, the organization Cribs for Kids has a national presence across the United States as well as a community engagement component where it has extended its presence across a range of community entities including healthcare systems through its hospitalwide certification program to promote infant sleep safety as a SIDS risk reduction measure.^6^ In addition, the national Safe-to-Sleep campaign in the United States has extended its visibility across both traditional and nontraditional formats to include virtual spaces and has engaged all of the fifty states across the United States in one or more ways through different community entities (e.g. local health departments, healthcare systems).^7^

 As part of heightening knowledge and awareness of SIDS worldwide, it is crucial to account for strategies that can cast a wider net in reaching more communities of infant caregivers as the basis to standardize SIDS reduction measures that are translatable across a range of community contexts. One communication medium that has risen in prominence during our ever-growing digital era is through social media.^8^ As long as individuals have access to technology, publicly present and archived information is oftentimes readily accessible via technology. In addition as national and global published research, campaigns, programs, and other initiatives are experiencing increased coverage on traditional and nontraditional sources of media, it is crucial to harness their potential into addressing some of the most pressing public health issues across the world.^9-11^ Infant mortality is certainly one of them. As a multitude of SIDS reduction measures are evidence-based, disseminating knowledge on them worldwide across digital spaces that extend beyond traditional sources of media could have a larger global impact and presence.

 Some of the most trending and widely accessible social media platforms for news uptake are YouTube, Facebook, Twitter and Instagram.^8,12^ Based on a recent review paper, evidence-based SIDS risk reduction measures were covered and examined across Facebook, Instagram, and Twitter.^9^ These studies presented several still images, tweets and exchange between group members on content pertaining to infant safe sleep positions, recommendations from a prior iteration of the AAP infant safe sleep guidelines in 2016, support of breastfeeding, avoidance of tobacco exposure and substance use, among more postnatal considerations for infant.^10,11,13^ Notably, none of these studies covered any prenatal content on risk factors for SIDS.

 To date, there has been no published study that has reviewed trending content pertaining to SIDS on YouTube. It follows that this study sought to fill this gap in SIDS awareness and reduction across the social media landscape. In turn, the goals of this descriptive observational study are the following: 1) describe sources, formats, and content of the widely viewed YouTube videos on SIDS; 2) assess whether content covered is in line with current SIDS reduction measures globally; and 3) present a range of clinical, public health and organizational recommendations for future research and practice.

## Methods

 The research design was cross-sectional in nature and involved collecting retrospective observational data at one conceptual point in time from the social media platform, YouTube. In December 2023 to January 2024, the researchers cleared the browser history on their computers. Equipped with a clean history on the computers, the researchers conducted a range of searches on YouTube that involved creating specific strings of key words. Piloting various key words (SIDS, sudden infant death syndrome, SUID, sudden unexpected infant death, crib death, cot death) was helpful in determining which phrase(s) yielded the most relevant videos, highest view counts across videos, and greatest cumulative views for the top 30, 60, and 100 videos, respectively. As part of screening, the researchers viewed videos across these pilot searches to assure that content pertained to SIDS and SUID. View counts below 10 000 were not accounted for in this sample, and thereby were not part of the most popular videos that constituted the 100 widely viewed ones. Notably, the search terms for “sudden unexpected infant death” and SUID yielded fewer relevant videos than “sudden infant death syndrome” and SIDS. It follows that the key words that formed the search strategy were “sudden infant death syndrome” which subsequently yielded the most widely viewed videos that were directly relevant to SIDS and SUID, likely attributed to SIDS constituting most of the SUID cases worldwide as aforementioned.^1^ The results were filtered by view count, and the URLs for the 100 most widely viewed videos were abstracted and safeguarded in a separate file. Overlapping URLs were deleted and replaced. Only one URL for each video was kept for coding.

 The researchers then created a codebook based on a review of literature and guidelines from authoritative agencies such as the AAP, Cribs for Kids, and the National Institutes of Health (NIH). The researchers viewed and coded all of the videos in this sample during December 2023 and January 2024. Inter- and intra-rater reliability of the coding was demonstrated. The following information was coded for each video: (a) source of upload, (b) format, (c) number of views, (d) length (in minutes), (e) year of upload, and (f) content.

###  Eligibility criteria

 One inclusion criterion in this study was that the videos reviewed would be published in English. Videos that did not have English narration or written content were excluded from the analysis. A second criterion was that the video focus primarily on SIDS and SUID. Each researcher viewed the full video, which constituted the unit of analysis. Music videos not centered on SIDS and SUID were also excluded. No requirements were specified based on length of time.

###  Measurements and coding specifications

 The instrument included the following basic information: coder, video identification number (which was assigned), date the video was uploaded, date the video was coded, length of video (in minutes), number of views, and title of the video. Following this general information, the instrument contained the following three sections: (a) source of upload, (b) format, and (c) content. Content included multiple variables (reviewed below), all of which were coded dichotomously (i.e., either yes or no) to indicate presence or absence in the respective video.

 The source of upload for each video was coded into one of the following four categories: organizational, consumer, governmental, and other sources. Organizational and governmental sources were professional in nature. Consumer sources involved the lay population. An example of an other source included a healthcare provider not affiliated with a healthcare system. The categories for coding format included Documentary; Interview; Demonstration/Experiment (e.g. modeling safe sleep practices, laboratory-based studies); Talk by Professional; TV Talk Show/Discussion panel; Animation; Still images; News report with anchor; V-blog; Advertisement; Testimonial/Story; Multiple formats; and “Other formats.” The content categories were formulated based on guidelines, recommendations, or clinically relevant anticipatory guidance from the AAP, Cribs for Kids, NIH, World Health Organization (WHO), and Healthy People 2030. A total of 14 content categories was created in this codebook: (a) illness and mortality (symptomology of SIDS); (b) compromised organs of infants; (c) environmental risk factors; (d) accessories, materials and resources; (e) AAP infant safe sleep guidelines; (f) community engagement; (g) caregivers involved in the care of infants; (h) timeframe; (i) high-risk health behaviors (e.g. tobacco, alcohol, substance use); (j) social determinants of health; (k) stressors and triggers; (l) psychiatric co-morbidities; (m) infant safe sleep care models; and (n) health benefits sin regard to infant development and survival. Conceptualization of the codebook involved developing these content categories to account for the totality of risk factors, considerations, as well as depth and breadth of targets for intervention surrounding SIDS risk reduction. AAP guidelines were accounted for in a separate category given their increased presence during pilot searches across the widely viewed videos. These guidelines are presented in Table 1.

**Table 1:** AAP infant safe sleep guidelines

**AAP Criteria**	**Level A Recommendations**
A-1	Back to sleep for every sleep
A-2	Use a firm flat noninclined sleep surface
A-3	Room share without bed share
A-4	No soft objects and loose bedding
A-5	Prenatal care
A-6	Avoid smoke exposure
A-7	Avoid alcohol and other drugs (during pregnancy and after)
A-8	Breastfeed
A-9	Offer pacifier
A-10	Avoid overheating and head covering
A-11	Do not use home monitors
A-12	Immunize per schedule
A-13	Promote tummy time
A-14	Endorsement and modeling by healthcare providers of infant safe sleep guidelines from time of prenatal care
A-15	Media and manufacturers follow safe sleep guidelines in their messaging and advertising to promote safe sleep practices as the social norm
A-16	Continue to promote components of the Safe-to-Sleep campaign
**AAP Criteria**	**Level B Recommendations**
B-1	Avoid use of commercial devices that are inconsistent with safe sleep recommendations
**AAP Criteria**	**Level C Recommendations **
C-1	Avoid swaddling
C-2	Continue research and surveillance on risk factors, causes and pathophysiological mechanisms of sleep-related deaths

###  Demonstration of intra- and inter-rater reliability

 The researchers demonstrated both the intra- and inter-rater reliability of the data in regard to the coding on the presence of content across the videos in this sample. To demonstrate intra-rater reliability, each researcher randomly selected 10 videos and recoded them within 2 weeks of the original coding. All of the dichotomously coded (Yes versus No) content variables in the instrument were accounted for in this analysis. Intra-rater reliability was found to be high (kappa = 0.95). Inter-rater reliability was demonstrated as well. Five videos were randomly selected from the 100 in the sample, and inter-rater agreement was also found to be high (kappa = 0.94).

###  Statistical analysis

 The analysis involved computing descriptive statistics for each of the variables examined in this study. Descriptive data pertaining to the characteristics of videos were summarized by calculating frequencies and percentages with respect to source, format, number of views, length, and content. For each content category, the number of videos that covered the content was identified. Next, the total number of views from the sample of videos covering each particular content element was subsequently determined. In addition, the proportion of total cumulative views was determined by dividing the number of views culminated by the specific videos covering each content element by the total cumulative views generated by all videos in this sample of widely viewed videos (N = 36 483 493 views). This analysis was conducted for all of the content categories specified in this study’s codebook via Statistical Package for the Social Sciences (SPSS), Version 29.0.2, Armonk, New York: IBM Corp.

 A limited dataset is also available for review on the statistical methods (descriptive statistics) as well as titles and weblinks of videos, their view counts at time of investigation, and length of each video. The collection and analysis method for the descriptive, observational data in this study complied with the terms and conditions for the source of this data.

## Results

 The total number of views for the sample of the 100 most widely viewed videos was 36 483 493. The view counts ranged from 10 220 to 6 788 347. These widely viewed videos were posted between 2006 to 2023. Length of videos ranged from 0.25 minutes to 38.4 minutes. The median length of the widely viewed videos was 2.28 minutes. The interquartile range for the sample ranged from 1.53 minutes to 3.40 minutes.

###  Sources

 A vast majority of the widely viewed videos originated from organizational/nongovernmental sources (N = 64), garnering greater than 25 million views and representing nearly 70% of the cumulative views. 17 videos were published by governmental sources and accounted for more than 2 million views (almost 7% of the cumulative views). 16 videos were posted by consumers, yielding greater than 8 million views and almost 24% of the cumulative views. Lastly, 3 videos were from other miscellaneous sources and attracted ~200 000 views (less than 1% of cumulative views).

###  Formats

 There was a range of different formats for the widely viewed videos. Talks by professionals were delivered in 42 videos, representing nearly 16 million views (~43% of the cumulative views). Demonstrations/experiments were covered in a comparative number of videos (N = 38) which accounted for almost 16 million views (also about 43% of the cumulative views). Testimonials/stories were a part of 21 videos, generating approximately 10 million views (~27% of the cumulative views). 20 videos were in other miscellaneous formats and represented greater than 9 million views, culminating in about 26% of the cumulative views. Still images were integrated among 40 videos, yielding almost 9 million views (~25% of the cumulative views). News reports with anchors were depicted in 17 videos which populated nearly 3 million views (almost 8% of the cumulative views). Animation was included in 14 videos, attracting almost 2 million views (about 5% of the cumulative views). 2 videos involved multiple formats, comprising greater than 100 000 views (less than 1% of the cumulative views).

###  Illness and mortality content 

 Four illness developments and fatal conditions were vastly represented in the widely viewed videos. These encompassed SIDS and were also referenced as contributing factors and sequalae of SIDS or near SIDS instances. SIDS was covered in 63 videos, attracting greater than 19 million views ( > 50% of the cumulative views). Mortality involved any reference or depiction of death among infant inclusive of SIDS or any other condition. Specifically, mortality was featured in 50 videos, culminating in > 16 million views (~46% of the cumulative views). Representation of respiratory compromise and suffocation included any reference or visualization of either one as a risk factor for SIDS. Examples of respiratory compromise were hypoxia, apnea, airway obstruction, respiratory distress and failure among more. There were 36 videos that integrated respiratory compromise, accounting for greater than 17 million views (about 48% of the cumulative views). Suffocation was covered in 52 videos, comprising more than 13 million views (37% of the cumulative views).

###  Content on environmental risk factors

 There was a wide range of representation of environmental risk factors across the widely viewed videos. Overheating was presented in 33 videos, culminating in nearly 16 million views (~44% of the cumulative views). Unsafe sleep positioning was integrated in 27 videos, attracting more than 15 million views (~43% of the cumulative views). Suffocation and strangulation risks were covered in 52 videos, garnering nearly 14 million views (37% of the cumulative views). Choking hazards were featured in 45 videos, yielding > 13 million views (~36% of the cumulative views). Co-sleeping / bed-sharing were portrayed in 41 videos, representing greater than 11 million views (~32% of the cumulative views). Tobacco exposure was depicted in 28 videos, generating more than 11 million views (almost 32% of the cumulative views). Loose bedding was integrated in 23 videos, accounting for approximately 9 million views (about 25% of the cumulative views). A comparative number of videos (N = 25) featured unsafe sleeping surfaces, similarly yielding ~9 million views (almost 25% of the cumulative views). Swaddling in the context of bundling or improper positioning was portrayed in 12 videos, constituting nearly 6 million views (~16% of the cumulative views). Substance and alcohol exposure was a part of 12 videos, comprising ~4 million views (about 10% of the cumulative views).

###  Content coverage of postnatal resources 

 There was also diverse representation of accessories, materials and resources reviewed across the widely viewed videos. Cribs were covered in 68 videos, attracting almost 28 million views and nearly 76% of the cumulative views. Bassinets were featured in 38 videos, garnering greater than 16 million views and approximately 45% of the cumulative views. Light clothes for infant while sleeping were covered in 33 videos, generating more than 13 million views and almost 37% of the cumulative views. Fitted cotton sheets were depicted in 49 videos, culminating in greater than 12 million views (~34% of the cumulative views). Although wearable blankets were portrayed in fewer videos (N = 23), there was a comparative number of views (~12 million) which accounted similarly for approximately 34% of the cumulative views.

###  Content on AAP infant safe sleep guidelines 

 The AAP infant safe sleep guidelines in prior and updated iterations were featured across the widely viewed videos in one or more ways. 69 videos presented content on infants sleeping on their backs, attracting nearly 25 million views (~70% of the cumulative views). 34 videos included content on room sharing without bedsharing, comprising more than 22 million views (~62% of the cumulative views). 59 videos covered content on avoiding soft objects and loose bedding (e.g. pillows, bumper pads, toys), garnering almost 22 million views (~60% of the cumulative views). Breastfeeding was integrated in 21 videos, yielding ~18 million views (approximately 50% of the cumulative views). 60 videos portrayed content on using a firm flat noninclined sleep surface, accounting for almost 18 million views (~50% of the cumulative views). Overheating and head covering were depicted in 33 videos, culminating in almost 16 million views (~44% of the cumulative views). Avoiding smoke exposure was reviewed in 28 videos, garnering greater than 11 million views (~32% of the cumulative views). Infants sleeping alone were featured in 23 videos, representing ~10 million views (about 27% of the cumulative views).

###  Community engagement content 

 There was also a diverse representation of community engagement from a range of stakeholders among the widely viewed videos. Healthcare systems were covered in 19 videos, garnering ~5 million views (about 13% of the cumulative views). Although police departments were only featured in one video, there were greater than 3 million views (nearly 10% of the cumulative views). The AAP was referenced in 17 videos, attracting > 3 million views (about 9% of the cumulative views).

###  Content coverage of caregivers 

 There was a wide dispersion in coverage of caregivers involved in the care of infants across the widely viewed videos. 16 of the widely viewed videos featured content pertaining to parents, garnering greater than 8 million views and nearly 23% of the cumulative views. Child care providers were included in 8 videos, accounting for ~1 million views (almost 3% of the cumulative views).

###  Prenatal and postnatal categorical content 

 All of the widely viewed videos covered content postnatally, populating greater than 36 million views (100% of the cumulative views). 18 videos featured prenatal content, accounting for greater than 8 million views (~22% of cumulative views). A comparative number of videos (N = 17) depicted content on the ABC Safe Sleep Model, yielding similarly greater than 8 million views and ~23% of the cumulative views.

###  Coverage of social determinants of health

 Social determinants of health in the context of their impact on SIDS received limited coverage across the widely viewed videos. Race/ethnicity was accounted for in 7 videos, yielding greater than 400 000 views (~1% of the cumulative views). Three videos included content related to gender, generating greater than 200 000 views (less than 1% of the cumulative views). Socioeconomic status was reviewed in two videos, garnering less than 200 000 views (also less than 1% of the cumulative views). Solely one video integrated content on access to healthcare, comprising greater than 100 000 views (less than 1% of the cumulative views).

###  Content on stressors and triggers

 A couple of stressors and triggers were depicted across the widely viewed videos. 10 videos covered content on caregiver fatigue, garnering almost 10 million views (approximately 27% of the cumulative views). Grief/loss was accounted for in 23 videos, representing greater than 10 million views (almost 28% of the cumulative views.

###  Content coverage of health benefits 

 Lastly, there was also variation in coverage of health benefits across the widely viewed videos. Increased safety was presented in 17 videos, representing greater than 7 million views and almost 20% of the cumulative views. Although infant development was covered in only 3 videos, a comparative 7 million views were generated among them which comprised also ~20% of the cumulative views. 8 videos featured content on decreased infant mortality, yielding almost 4 million views and about 10% of the cumulative views. Tables 2–6 present a comprehensive breakdown of number of views and cumulative views for sources, formats and content among the widely viewed videos on SIDS knowledge and reduction.

**Table 2:** Frequencies, view counts, and cumulative view count percent of widely viewed SIDS videos by upload source

**Classification of the source of video upload**	**N**	**View count **	**Cumulative view count percent (%)**
Organizational/Nongovernmental	64	25 241 557	69.19
Consumer	16	8 590 336	23.55
Governmental	17	2 420 669	6.63
Other	3	230 931	0.63

**Table 3:** Frequencies, view counts, and cumulative view count percent of widely viewed SIDS videos by format

**Format**	**N**	**View count **	**Cumulative view count percent (%)**
Talk by professional	42	15 927 088	43.66
Demonstration/Experiment	38	15 766 306	43.21
Testimonial/Story	21	9 865 865	27.04
Other formats	20	9 363 775	25.67
Still Images	40	8 967 249	24.58
News report with anchor	17	2 913 183	7.98
Animation	14	1 877 908	5.15
Multiple formats	2	141 842	0.39

Note: More than one response is possible across videos.

**Table 4:** Frequencies, view counts, and cumulative view count percent of widely viewed sids videos by illness/mortality

**Illness/Mortality **	**N**	**View count **	**Cumulative view count percent (%)**
SIDS	63	19 445 446	53.3
Respiratory compromise	36	17 533 800	48.06
Mortality	50	16 644 344	45.62
Suffocation	52	13 486 466	37
SUID	8	1 339 099	3.67
Asphyxiation	4	1 294 222	3.55
Strangulation	6	887 842	2.43
Entrapment	10	731 760	2
Prematurity	7	446 864	1.22
Low Birth Weight	6	421 766	1.16
Aspiration	1	59 961	0.16

SIDS, Sudden Infant Death Syndrome; SUID, Sudden unexpected infant death. Note: More than one response is possible across videos.

**Table 5:** Frequencies, view counts, and cumulative view count percent of widely viewed SIDS videos by safe sleep materials, accessories, and resources

**Safe sleep materials, accessories, and resources**	**N**	**View count**	**Cumulative view count percent (%)**
Cribs	68	27 768 953	76.11
Bassinet	38	16 465 783	45.13
Light clothes for infant while sleeping	33	13 311 021	36.49
Fitted cotton sheets	49	12 530 226	34.34
Wearable blanket	23	12 412 938	34.02
Pacifiers	21	5 990 414	16.42
Sleep sacks	25	5 561 468	15.24
Pack-N-Plays	13	2 208 522	6.05
Baby beds	2	2 184 251	5.99
Baby monitors	5	1 876 544	5.14
Infant sleep boxes	5	1 362 433	3.73
Play yards	8	1 124 853	3.08
Co-sleeper	11	1 045 741	2.87
Books	1	637 928	1.75
Basket	1	123 746	0.34

Note: More than one response is possible across videos.

**Table 6:** Frequencies, view counts, and cumulative view count percent of widely viewed SIDS videos by AAP safe sleep guidelines

**AAP safe sleep guidelines **	**N**	**View count **	**Cumulative view count percent (%)**
Back	69	24 743 248	67.82
Room share without bed share	34	22 449 376	61.53
No soft objects and loose bedding (e.g. pillows, bumper pads, toys)	59	21 574 000	59.13
Breastfeed	21	18 131 703	49.7
Use a firm flat noninclined sleep surface	60	17 709 559	48.54
Overheating and head covering	33	15 972 181	43.78
Avoid smoke exposure	28	11 560 591	31.69
Alone	23	9 830 080	26.94
Avoid alcohol and other drugs (during pregnancy and after)	12	3 745 412	10.27
Promote tummy time	11	1 507 314	4.13
Avoid use of commercial devices that are inconsistent with safe sleep recommendations	10	1 292 776	3.54
Offer pacifier	10	1 273 575	3.49
Media and manufacturers follow safe sleep guidelines in their messaging and advertising to promote safe sleep practices as the social norm	4	977 794	2.68
Prenatal care	7	601 156	1.65
Immunize per schedule	4	549 111	1.51
Continue research and surveillance on risk factors, causes, and pathophysiological mechanisms of sleep-related deaths	13	523 723	1.44
Continue to promote components of the Safe-to-Sleep campaign	5	465 406	1.28
Endorsement and modeling by healthcare providers of infant safe sleep guidelines from time of prenatal care	2	444 114	1.22
Do not use home monitors	2	344 537	0.94
Avoid swaddling	2	151 265	0.41

Note: More than one response is possible across videos.

## Discussion

 The top eight videos cumulatively generated greater than 24 million views out of the nearly 36 million views for this study’s sample of 100 widely viewed videos on SIDS reduction. None of them were published after the 2022 revised AAP infant safe sleep guidelines. Many of the videos supported the AAP guidelines, heightening reliability and credibility of content reviewed. However, none of the videos presented any specific guidelines delineated by any other national or global organization. Healthcare systems, police departments, and the AAP were community and national groups that were widely represented, each generating > 3 million views. Videos posted by nongovernmental organizations (N = 64) attracted the most views ( > 25 million). Talks by Professionals and Demonstrations/Experiments garnered the largest view counts ( > 30 million total). Four illnesses and fatal conditions (SIDS, respiratory compromise, mortality, and suffocation) were vastly represented across the widely viewed videos. Multiple postnatal environmental risk factors were widely covered. Notably, social determinants of health received limited coverage (cumulatively < 1 million views). Prenatal risk factors for SIDS were not widely covered, generating less than a quarter of the total views. Grief/loss and caregiver fatigue were largely reviewed, each garnering ~10 million views. Mood and anxiety disorders (including postpartum depression) were not covered. Several health benefits were largely examined including increased safety of infant, optimization of infant development, and decreased infant mortality. The ABC Safe Sleep Model and Triple Risk Model for SIDS received minimal coverage. Notably, resources depicted solely pertained to accessories in the infant safe sleep environment.

###  Prenatal Implications

 As aforementioned, most of the widely viewed videos presented content more relevant to the postnatal timeframe and the only infant harm reduction care model that received coverage across the widely viewed videos pertained to the ABC Safe Sleep Model. There are several modifiable and nonmodifiable risk factors for SIDS that emerge prenatally which received limited coverage across these videos. Of note, the Triple Risk Model for SIDS accounts for prenatal risk factors; however, there were no videos in this sample that covered this. As the risk of SIDS begins as early as the prenatal stage, it is crucial to increase visibility of this information and endorsement for addressing them on a continuum from prenatal to postnatal care. Given the substantial emphasis on environmental risk factors postnatally among these videos, it is crucial to also equitably account for the ones present prenatally that the Triple Risk Model for SIDS covers to optimize addressing SIDS on a continuum from time of pregnancy to post-birth. Integrating content on prenatal environmental risk factors, prenatal care, and the Triple Risk Model for SIDS could help heighten awareness on the constellation of considerations prenatally that could contribute towards SIDS risk reduction. One key consideration is to utilize videos that are easy to access for families of infants. It follows that disseminating these videos (especially the top 8) to prenatal clinics and further to prenatal parenting classes could be vital in heightening the initiation of SIDS reduction education from the prenatal phase onward.

###  Organizational implications

 The widely viewed videos were in adherence with several of the AAP infant safe sleep guidelines published before the 2022 iterations. These findings highlight their reliability in following organizational guidelines in the USA, thereby further strengthening their credibility for inclusion in patient and family education. Notably, none of the videos presented any specific guidelines outlined by any other national or global organization, although resources from a couple of countries in Europe were featured among the widely viewed videos. Future coverage of videos could account for any of the recommendations or guidelines published by other national and global organizations and countries worldwide as the basis to support common elements across SIDS reduction measures as universal recommendations in contributing towards addressing SIDS as a leading cause of infant mortality.

###  Implications for healthcare providers

 A vast majority of the widely viewed videos were published by nongovernmental / organizational sources that included healthcare systems, the AAP and Cribs for Kids. These findings are similar to different cross-sectional studies that examined health-related content on YouTube posted by primarily academic, nongovernmental sources.^14-16^ Many of the videos in this study involved discussions by healthcare providers who also provided demonstrations in the optimization of infant’s sleep environment. This greater representation of content provided by healthcare communities also delimits dissemination of misinformation as each deliverer of care is more likely to follow infant safety guidelines including the safe sleep recommendations provided by the AAP in the United States or as endorsed in other countries. In a couple of European countries, healthcare providers were actively involved in providing education on infant sleep boxes for infants coming home from the hospital for the first time to promote infants sleeping alone on their backs in a separate environment which suggests that it is likely a universal recommendation for infant sleep environments worldwide. It follows that healthcare providers could also increase their visibility in addressing modifiable risk factors for SIDS prenatally as the basis to support and disseminate knowledge and awareness globally of SIDS reduction measures on a continuum.

 From a health equity perspective, it is crucial to assure equitable access to healthcare services and resources for prenatal and postnatal care pertaining to SIDS reduction education. Notably, the total number of views among the widely viewed videos in this study (~36 million) was substantially higher than the cumulative views across different cross-sectional studies examining health related content on YouTube^14,15,17,18^ but lower than the cumulative views garnered in other cross-sectional studies.^19-24^ Given the large following overall on YouTube from the constellation of these studies, YouTube yields promise for consumer health education across the healthcare landscape. It follows that in the meantime, healthcare providers could direct caregivers to available resources on SIDS reduction education (e.g. via YouTube) to review on their own time.

###  Community engagement considerations

 Notably, social determinants of health received scant coverage across the widely viewed videos. Social determinants of health comprise either risk or protective factors for SIDS and ultimately are a predictor of a family’s access to a range of infant safe sleep environmental resources postnatally as well as access to prenatal care. In fact, many of the recommendations in the AAP infant safe sleep guidelines will only extend as far as a family’s access to the resources to meet these recommendations for their infants. The built environment and support networks form the heart of community engagement in reaching the community. However, many of the key institutional stakeholders and entities (e.g. faith-based organizations, community centers, fire departments, and libraries) were not covered in the widely viewed videos. This finding is similar to the limited coverage of social determinants of health.

 Community engagement to increase equitable access to resources in a community can also contribute towards heightening social determinants of health as strengths for a family in navigating prenatal care and securing a safe sleep environment at home for their infants postnatally. Given that several of the widely viewed videos were in line with AAP guidelines, one direction to reach more families of infants in the community could involve disseminating these videos to not only prenatal clinics and parenting classes but also to pediatric primary care clinics, family medicine practices, WIC centers, and healthcare systems. Future community stakeholders could also increase their presence on social media to heighten awareness of ways that they have and could contribute towards improving access to resources for caregivers of infants on a continuum from prenatal to postnatal stages as a promising direction in reducing the risk of SIDS, thereby further contributing towards decreasing infant mortality.

###  Governmental content considerations 

 Surprisingly, there was limited representation of content published by any government across the widely viewed videos. Infant mortality remains a longstanding public health epidemic and there is implication that the government shares responsibility in mitigating this crisis. One notable public health campaign, the Safe-to-Sleep campaign in the USA was not widely covered and no other campaign or initiative spearheaded by the government in any other countries was represented in this sample of videos. It is imperative to find ways to make governmental content more appealing, relatable, and findable so that it is viewed more often across the world. Perhaps increased engagement of the government with nongovernmental organizations and consumers could heighten content creation that is more engaging, acceptable, and in line with the preferences among caregivers of infants worldwide.

###  Clinical care implications

 Given the findings of this study, it is possible that consumers worldwide are reached more by these videos than by healthcare providers. For example in the USA alone, there are approximately 11 million infants across the country and the total number of views for these 100 videos was nearly triple the population estimate (~36 million views). One key question that remains is whether social media has the potential to change caregiver practices in SIDS reduction. However, the data for SIDS reduction video utilization is not yet sufficiently compelling for them to be included as part of evidence-based patient and family education or clinical practice guidelines at this point in time.

 In addition, SIDS prevention messages have consistently demonstrated effectiveness when integrated into conversations with families as evidenced by prior studies on health promotion in the SIDS reduction landscape through safe sleep education across community and healthcare settings.^25-31^ It follows that harnessing the potential of widely viewed messaging across these videos for inclusion in discussions between families of infants and healthcare providers across prenatal, primary and inpatient care settings could be an opportunity to assess the efficacy of integrating this content into clinical practice.

 Across the sample of videos in this study, most of them presented content on illness and mortality considerations, either in the representation of SIDS, SUID and one or more medical complications (e.g. respiratory compromise in one or more ways) arising from an adverse event (e.g. co-sleeping with infant face-down) that could result from an unsafe infant sleep practice or from nonmodifiable risk factors (e.g. prematurity, low birth weight). Other complications covered pertained to preventable morbid circumstances (e.g. suffocation, strangulation, asphyxiation). These videos generated a larger number of views, suggesting that this content yielded increased engagement among viewers which could be attributed to their fear-based appeals as well as clinical relevance. In addition, several of the widely viewed videos featured testimonials of caregivers who had lost an infant in combination with still images. These videos similarly generated a greater number of views, suggesting that these shared narratives heighten engagement given their emotionally loaded content that could further increase perceived severity, susceptibility, and relatability among a range of caregivers involved in the care of infants. It follows that future content from social media coverage of SIDS integrate continued considerations for illness and mortality alongside addressing grief and loss from stories of caregivers who have lost their infants to SIDS.

###  Inclusivity of caregivers

 In addition although parents were referenced in several of the widely viewed videos, additional caregivers who are oftentimes involved in the care of infants (e.g. child care providers, grandparents, other extended family members) received minimal representation among the videos. It is crucial to account for the totality of caregivers and heighten SIDS reduction education for all of them given that postnatal environmental risk factors for SIDS could be ever present in the care of any caregiver of the infant, especially when the infant is asleep. Several community and national initiatives (e.g. across local health departments and the national Safe-to-Sleep campaign) are expanding their awareness and outreach efforts to account for the range of caregivers as the basis to have a larger clinical impact in addressing preventable behavioral risk factors for SIDS. It follows that these community and national initiatives could extend their presence on social media to heighten knowledge and awareness on the significance of involving and reaching all caregivers of infants worldwide as the basis to disseminate knowledge on ways that they could contribute towards saving the lives of infants.

## Limitations

 This study was limited in several significant ways that must be considered when interpreting the results and conclusions. First, the design of the present study was cross-sectional, which certainly limits generalizability of findings over time. This is particularly important given the ever-evolving nature of the videos on YouTube, and the views they attract are changing constantly. A second limitation pertained to the sample reviewed in this study. Although the videos were selected after clearing browser history, the algorithm that generated the resulting sample remains unknown. It is possible that some of the widely viewed videos were not included at this conceptual point in time. In addition, delimiting the scope to videos in English further limits generalizability, which is important since there is substantial variation in SIDS rates across the world where English is not the primary language. Also, the sample size of 100 videos was arbitrary. A third limitation is related to the descriptive and observational nature of the data themselves. The main outcome in this study was number of views, as based on the premise that reach and engagement are important ways to assess health communications; in turn, no other engagement metric (e.g. likes, comments) were reviewed. However, there was no way to distinguish between number of views versus number of viewers. Another issue was that the findings pertaining to content coverage were disproportionately influenced by a comparatively small number of videos that attracted a comparatively large proportion of views. A fourth limitation involved the limitations surrounding the search strategy. It is possible that substantially fewer videos among the widely viewed covered prenatal considerations (e.g. prenatal care, nonmodifiable risk factors present from prenatal timeframe) given the inherent nature of the search strategy. Despite these limitations, the findings are significant for several reasons. The videos in this study received over 36 million views, suggesting that people are searching YouTube to learn about ways to address risks of SIDS. Given this wide reach, descriptions of information that are and are not being conveyed are crucial for public health education and promotion.

## Conclusion

 SUID remains one of the leading causes of infant mortality worldwide, largely driven by SIDS. This is the first study to date that examined SIDS coverage among the widely viewed videos on a trending and popular social media platform, YouTube. Many of the videos involved heightening knowledge and awareness of a multitude of risk factors for SIDS and provided recommendations on infant safe practices and resource utilization. Notably, there were substantially fewer videos that covered prenatal considerations. It follows that addressing the wide range of risk factors for SIDS on a continuum from time of pregnancy to post-birth could support infant safety, reduced infant mortality, and overall healthier development of the infant, especially during the first critical year of life where the risk of SIDS is substantially higher among infants. National and global health organizations could take a more active presence on disseminating knowledge of SIDS reduction measures in combination with credible delivery of content from healthcare providers across these organizations alongside emotionally laden testimonials of caregivers grieving the loss of their infants attributed to SIDS. The integration of these approaches could spark up increased engagement that could be further assessed in future health behavior change among caregivers of infants as well as expectant caregivers as targets for future interventions in SIDS reduction.

## Completing Interests

 The authors declare that they have no competing financial interests.

## Data Availability Statement

 All of the data from our study is publicly available on the social media platform, YouTube.

## Ethical Approval

 Not Applicable.
